# Testimoniale Ungerechtigkeit gegenüber Menschen mit psychischer Erkrankung in der Gesundheitsversorgung. Eine konzeptionelle und ethische Analyse

**DOI:** 10.1007/s00481-021-00666-7

**Published:** 2021-11-16

**Authors:** Mirjam Faissner, Georg Juckel, Jakov Gather

**Affiliations:** 1 Klinik für Psychiatrie, Psychotherapie und Präventivmedizin, LWL-Universitätsklinikum, Ruhr-Universität Bochum, Alexandrinenstraße 1–3, 44791 Bochum, Deutschland; 2 Institut für Medizinische Ethik und Geschichte der Medizin, Ruhr-Universität Bochum, Markstraße 258a, 44799 Bochum, Deutschland

**Keywords:** Ableismus, Diskriminierung, Psychiatrie, Gesundheitssystem, Epistemische Ungerechtigkeit, Ableism, Discrimination, Psychiatry, Health care system, Epistemic injustice

## Abstract

Menschen mit psychischer Erkrankung sterben statistisch gesehen früher als die Allgemeinbevölkerung. Ein Grund hierfür ist, dass sie eine schlechtere somatische Gesundheitsversorgung erhalten. Wir argumentieren, dass ableistische Netzwerke sozialer Bedeutung zu einer Abwertung der epistemischen Kompetenz von Menschen mit psychischer Erkrankung führen. Diese Abwertung kann mit dem Konzept der testimonialen Ungerechtigkeit erfasst werden. Testimoniale Ungerechtigkeit bezeichnet das ungerechtfertigte Herabstufen der Glaubwürdigkeit einer*s Sprecher*in aufgrund eines Vorurteils gegen ihre*seine soziale Identität. Wir analysieren ethische und epistemische Folgen testimonialer Ungerechtigkeit als wichtige Ursachen der schlechteren Gesundheitsversorgung von Menschen mit psychischer Erkrankung. Testimoniale Ungerechtigkeit kann zu medizinischen Behandlungsfehlern führen und für Betroffene schwerwiegende gesundheitliche Folgen nach sich ziehen. Zudem kann sie zu einem Vertrauensverlust von Menschen mit psychischer Erkrankung in das Gesundheitssystem führen. Daher trägt testimoniale Ungerechtigkeit zur strukturellen Diskriminierung von Menschen mit psychischer Erkrankung bei. Vor diesem Hintergrund diskutieren wir, wie die somatische Gesundheitsversorgung unter ethischen Gesichtspunkten verbessert werden kann.

## Abstract (en)

**Definition of the problem:**

Statistically, people with mental disorders die prematurely due to preventable and treatable somatic illness. We analyze testimonial injustice as one mechanism which contributes to poor quality of health care for people with mental disorders.

**Argument:**

Practices in somatic health care are structured by ableist networks of social meaning. This leads to a systemic downgrading of the epistemic capacities of people with mental disorders and to a disproportionate focus on psychic disability and mental disorder in diagnostic processes. As a consequence, people with mental disorders are at high risk of suffering testimonial injustice in somatic health care. Testimonial injustice refers to the unjustified downgrading of a speaker’s credibility based on a prejudice against their social identity. Testimonial injustice has important epistemic and ethical implications. It can lead to medical errors with serious health consequences for those affected. It can also lead to a loss of trust in the health system by people with mental disorders.

**Conclusion:**

Testimonial injustice against people with mental disorders reproduces structural discrimination and is ethically problematic. Practices of somatic health care need to be improved to reduce discrimination.

## Einleitung

Menschen mit schwerer psychischer Erkrankung sterben statistisch gesehen etwa 10 bis 20 Jahre früher als die Allgemeinbevölkerung aufgrund behandelbarer somatischer Erkrankungen (Juckel 2014; Schneider et al. 2019). Diese Tendenz zeigt sich auch in der COVID-19-Pandemie mit einer signifikant erhöhten Mortalität von Menschen mit psychischer Erkrankung (Wang et al. 2020). Neben Faktoren auf individueller, institutioneller und struktureller Ebene nimmt die WHO (2015) als ursächlich an, dass somatische Beschwerden von Menschen mit psychischer Erkrankung in somatischen Versorgungssettings überproportional häufig ignoriert werden.

In diesem Artikel vertreten wir die These, dass Menschen mit psychischer Erkrankung in somatischen Behandlungssettings aufgrund ableistischer Netzwerke sozialer Bedeutung einem erhöhten Risiko für testimoniale Ungerechtigkeit unterliegen.^1^ Testimoniale Ungerechtigkeit bezeichnet das ungerechtfertigte Herabstufen der Glaubwürdigkeit einer Person aufgrund eines negativen Vorurteils gegen ihre soziale Identität (Fricker 2007). Zunächst einige begriffliche Vorbemerkungen: In Anlehnung an die Definition der *World Health Organisation *(WHO 2019) verstehen wir psychische Erkrankung als Veränderungen des Denkens, der Wahrnehmung, der Emotionalität, des Verhaltens und der Beziehungsbildung mit einhergehendem Leiden, die im medizinischen Setting mittels standardisierter Diagnosen erfasst werden. Psychische Erkrankung kann mit Behinderung (*disability*) einhergehen. Wie in den *Disability Studies* etabliert, unterscheiden wir dabei zwischen in Veränderungen des psychischen Erlebens verorteten Einschränkungen (*impairment*) und durch soziale Barrieren verursachte Behinderung der sozialen Partizipation.^2^ Der Begriff *Ableismus* (abgeleitet aus dem Englischen *able* = fähig) bezeichnet Praktiken und Netzwerke sozialer Bedeutung, in denen ein idealisierter und als frei von Einschränkungen konstruierter Körper als Norm gesetzt wird, während Körper mit Einschränkungen diskriminiert und unsichtbar gemacht werden. Wir wählen einen *Disability Studies* Ansatz, da dieser zum einen die Sichtbarmachung von sozial bedingter Benachteiligung von Menschen mit psychischer Erkrankung erlaubt und zum anderen auf die durch die UN-Behindertenrechtskonvention (UN-BRK) verbrieften Menschenrechte von Menschen mit psychischer Behinderung verweist.

Unser Vorgehen ist wie folgt strukturiert. Zunächst stellen wir das Konzept der epistemischen Ungerechtigkeit vor und analysieren die medizinische Praxis als epistemische Praxis. Anschließend erfolgt eine Einführung des Konzepts der testimonialen Ungerechtigkeit anhand klinischer Fallbeispiele. Basierend auf empirischen Untersuchungen zu Bias und Vorurteil in der medizinischen Praxis zeigen wir, inwiefern Menschen mit psychischer Erkrankung einem besonderen Risiko ausgesetzt sind, testimoniale Ungerechtigkeit zu erfahren. Dabei identifizieren wir ableistische Netzwerke sozialer Bedeutung als grundlegend. Diese beinhalten eine Abwertung von normabweichenden Sprech- und Denkweisen, wie sie im Rahmen psychischer Erkrankungen auftreten können, und bergen daher ein hohes Risiko für testimoniale Ungerechtigkeit. Wir analysieren ethische sowie epistemische Folgen testimonialer Ungerechtigkeit im Kontext der medizinischen Praxis. In unserer Analyse stellt testimoniale Ungerechtigkeit, erstens, ein intrinsisches moralisches Problem dar, da Menschen in ihrer Fähigkeit als Wissende, und damit in einer zentralen Eigenschaft als Menschen, geschädigt werden. Zweitens zieht testimoniale Ungerechtigkeit epistemisch und ethisch bedeutsame Folgen mit sich, die für die Gesundheitsversorgung relevant sind. Testimoniale Ungerechtigkeit kann zu einem Vertrauensverlust in eigene Fähigkeiten sowie in das medizinische System führen. Auch kann es zu epistemischen Fehlern im Prozess des Diagnostizierens und dadurch zu ernsthaften medizinischen Fehlern führen. Auf dieser Grundlage argumentieren wir, dass Menschen mit psychischer Erkrankung gegenüber Menschen ohne psychische Erkrankung einen Nachteil *qua* ableistischer Diskriminierung erfahren. Abschließend diskutieren wir Strategien der Vermeidung testimonialer Ungerechtigkeit in somatischen Versorgungssettings für Menschen mit psychischer Erkrankung.

## Epistemische Ungerechtigkeit

### Medizinische Praxis als epistemische Praxis

Seit der Veröffentlichung von Frickers (2007) Werk *Epistemic Injustice – Power & the Ethics of Knowing* hat das Gebiet der epistemischen Ungerechtigkeit vermehrt Aufmerksamkeit erfahren. Gegenüber der Annahme, dass Wissen primär epistemischen Normen unterliegt, wurde argumentiert, dass Praktiken der Wissensproduktion und -dissemination fundamental sozial sind. Zugrunde liegt, dass Individuen miteinander als epistemische Agent*innen interagieren, also als Individuen, die sich Wissen oder Verständnis aneignen und darüber Zeugnis geben können (Puddifoot 2018). Als soziale Praktiken können epistemische Praktiken Machtdynamiken involvieren und Schauplatz sozialer Ungerechtigkeiten werden. Daher sind sie ethisch bedeutsam und unterliegen neben epistemischen auch ethischen Normen. Epistemische Ungerechtigkeit bezeichnet Formen der Ungerechtigkeit, die Individuen in ihrer Fähigkeit als epistemische Agent*innen erfahren können. Fricker unterscheidet zwei zentrale Formen epistemischer Ungerechtigkeit: testimoniale Ungerechtigkeit, bei der eine Person aufgrund negativer Stereotype nicht als epistemische*r Agent*in anerkannt wird, und hermeneutische Ungerechtigkeit, bei der Menschen von Praktiken der Wissensproduktion ausgeschlossen und damit epistemisch marginalisiert werden. Theorien epistemischer Ungerechtigkeit(en) wurden in den letzten Jahren theoretisch weiterentwickelt und auf unterschiedliche Bereiche, wie die medizinische Praxis (Kidd und Carel 2017) oder die empirische Bioethik (Schicktanz 2012) angewendet. Im Folgenden konzentrieren wir uns auf testimoniale Ungerechtigkeit gegenüber Menschen mit psychischer Erkrankung im medizinischen Setting.^3^

Inwiefern sind epistemische Praktiken für das medizinische Setting relevant? Die Interaktion zwischen Behandler*innen und Menschen, die medizinische Versorgungsdienste nutzen (im Folgenden „Nutzer*innen“ genannt), ist primär praktisch-kommunikativ. Nach einem deliberativen Modell erarbeiten Nutzer*innen und medizinisches Personal gemeinsam eine Lösung für ein medizinisches Anliegen (sog. partizipative Entscheidungsfindung) (Krones 2015). Basierend auf Anamnese und Untersuchung werden verschiedene Differentialdiagnosen, die die Beschwerden der Person am ehesten erklären können, in Betracht gezogen. Um eine Diagnose zu bestätigen und Alternativen auszuschließen, werden diagnostische Untersuchungen durchgeführt. Der vierstufige Prozess – Anamnese und Untersuchung – Erstellung von Differentialdiagnosen – spezifische diagnostische Maßnahmen – Behandlungskonzept stellt eine typische, problemorientierte medizinische Arbeitsweise dar. Dabei handelt es sich um eine epistemische Praxis, deren zentraler Bestandteil ist, im Rahmen eines kollaborativen Prozesses zwischen Nutzer*innen und medizinischem Personal, Informationen zu teilen und Verständnis zu generieren.

### Testimoniale Ungerechtigkeit

In den letzten Jahren hat sich eine Fülle von Arbeiten damit auseinandergesetzt, wie Nutzer*innen als epistemische Agent*innen in der medizinischen Praxis Ungerechtigkeit erfahren können (Kidd und Carel 2017). Betrachten wir dazu folgende Fallvignetten:

#### Fall 1

Bei einem Nutzer mit schwerer psychischer Erkrankung und Diabetes mellitus wird eine fortgeschrittene diabetische Polyneuropathie (eine durch Diabetes verursachte Schädigung von peripheren Nerven) diagnostiziert. Er gibt an, Beschwerden in den Füßen schon seit fünf Jahren regelmäßig gegenüber seinen Behandler*innen berichtet zu haben. *(Fall aus qualitativer Studie von Nash (* 2014, S. 719–720), basierend auf den Aussagen von Studienteilnehmer Nr. 4, adaptiert und übersetzt durch die Autor*innen).

#### Fall 2

Eine Nutzerin auf einer psychiatrischen Station zur Therapie einer Borderline-Persönlichkeitsstörung klagt über wiederkehrende Oberbauchschmerzen, die anders seien als ihr vorbekannte Stress-induzierte Bauchschmerzen. Die Beschwerden werden zunächst nicht weiter abgeklärt, da die Behandler*innen von einer psychosomatischen Genese ausgehen. Während der nächsten drei Wochen gibt die Nutzerin anhaltend starke Schmerzen an. Schließlich erfolgt eine internistische Abklärung, bei der eine bakterielle Entzündung der Magenschleimhaut festgestellt wird, die antibiotisch behandelt wird. *(Fall aus eigener klinischer Praxis).*

In beiden Fällen berichten die Nutzer*innen über Symptome, die als medizinisch abklärungsbedürftig einzuschätzen sind. Dabei stoßen sie auf Schwierigkeiten, sich bezüglich ihrer Beschwerden Gehör zu verschaffen. Basierend auf zahlreichen Berichten von Nutzer*innen argumentieren Kidd und Carel (2017), dass solche Fälle systematisch vorkommen und schlagen vor, diese mit dem Konzept der testimonialen Ungerechtigkeit zu erklären. Fricker (2007, S. 28) definiert testimoniale Ungerechtigkeit als ein negatives Glaubwürdigkeitsurteil aufseiten der zuhörenden Person über die sprechende Person aufgrund eines negativen Vorurteils gegen die soziale Identität der sprechenden Person. Gemäß ihrer Analyse prüfen epistemische Agent*innen die Relevanz und Überzeugungskraft des Gesagten, etwa um beurteilen zu können, ob es ihnen hinreichenden Grund gibt, ihr eigenes Überzeugungssystem anzupassen (Fricker 2007, S. 36). Bei der Wahrnehmung der Glaubwürdigkeit, also der Kompetenz und Ehrlichkeit, einer*s Sprecher*in spielen Stereotype eine wichtige Rolle. Ein Stereotyp bezeichnet eine Generalisierung über eine soziale Gruppe in Form einer verbreiteten Assoziation zwischen der Gruppe und einem oder mehreren Attributen (Fricker 2007, S. 30). Es kann entweder explizit berücksichtigt werden oder automatisch und unbewusst in der Form eines impliziten Bias in die Urteilsbildung einfließen (Saul 2017). Laut Fricker können Stereotype verlässliche Heuristiken bei der Bildung sozialer Urteile darstellen, so dass Personen auch bei unvollständiger Kenntnislage effektiv Urteile über die Glaubwürdigkeit einer Person fällen können. Beispielsweise ist das Stereotyp, dass sich Virolog*innen gut mit viralen Infektionskrankheiten auskennen, eine sinnvolle Heuristik, um die Ausführungen einer Virologin zur COVID-19-Pandemie als *prima facie* kompetent und ehrlich einzuschätzen. Manche Stereotypisierungen beruhen jedoch auf epistemisch dysfunktionalen Vorurteilen, die ohne einen adäquaten Einbezug von verfügbarer Evidenz gefällt werden:

A widely held disparaging association between a social group and one or more attributes, where this association embodies a generalization that displays some (typically, epistemically culpable) resistance to counter-evidence owing to an ethically bad affective investment. (Fricker 2007, S. 35)

Wird die Glaubwürdigkeit einer*s Sprecher*in aufgrund eines solchen negativen Stereotyps abgewertet, handelt es sich um testimoniale Ungerechtigkeit, da die Person, beruhend auf Vorurteilen gegenüber ihrer sozialen Identität, in der Ausübung einer zentralen epistemischen Aktivität untergraben wird. Relevant ist, dass die Stereotype nicht Teil der bewussten Überzeugungssysteme der zuhörenden Person sein müssen, sondern implizit das Glaubwürdigkeitsurteil beeinflussen können (Fricker 2007, S. 36).

### Stereotype und Bias in der medizinischen Versorgung

Der Einfluss von Stereotypen und unbewussten Bias auf medizinisches Personal und medizinische Praktiken ist empirisch gut belegt und hat unterschiedliche Auswirkungen auf die Wahrnehmung und Interpretation von Symptomen, den Kommunikationsstil oder die Durchführung diagnostischer Maßnahmen (Cooper et al. 2012; White 2014; Hall et al. 2015). So werden von als Frauen oder nicht-*weiß* gelesenen Personen angegebene Schmerzen systematisch als weniger ernst eingestuft als die Schmerzen von *weiß* und männlich gelesenen Personen, es werden andere Differentialdiagnosen in Betracht gezogen und Schmerzmittel später und zurückhaltender eingesetzt (Hoffmann und Tarzian 2001; Werner und Malterud 2003). Dies impliziert, dass bei dem oben skizzierten diagnostischen Prozess nicht nur zählt, welche Beschwerden angegeben werden, sondern auch, wer spricht, d. h. welche soziale Identität der*die Nutzer*in hat.

Der Einfluss von Stereotypen und impliziten Bias steht *prima facie* im Widerspruch zu den von Ärzt*innen angestrebten egalitären Prinzipien der Unvoreingenommenheit (FitzGerald und Hurst 2017). Ein Grund für diese Diskrepanz zwischen normativen Idealen und Praxis kann in der Internalisierung von vorurteilsbehafteten Netzwerken sozialer Bedeutung liegen. Aufgrund der Komplexität sozialer Strukturen ist es von Vorteil, wenn Personen soziale Bedeutungen internalisieren, so dass sie automatisch an kooperativen sozialen Praktiken teilnehmen können (Haslanger 2017). Dabei bezeichnen Netzwerke sozialer Bedeutung intersubjektive Wahrnehmungs‑, Denk- und Verhaltensmuster, die Theorien, Konzepte, Hintergrundannahmen und Stereotype, praktisches Wissen sowie soziale Skripte für die Interaktion mit unserem sozialen Umfeld beinhalten. Haslanger (2017) argumentiert, dass diese verzerrt sein können und beispielsweise sexistische oder rassistische negative Stereotype und diskriminierende soziale Skripte enthalten können, die zu struktureller Diskriminierung beitragen. Derartige Verzerrungen sind Teilnehmer*innen in sozialen Praktiken nicht notwendigerweise bewusst, da häufig nicht offensichtlich ist, welche Auswirkungen soziale Praktiken in komplexen sozialen Strukturen haben. Folglich setzt eine Ungleichbehandlung aufgrund verzerrter Netzwerke sozialer Bedeutung keine Absichtlichkeit voraus. Dies ist für den Bereich der Gesundheitsversorgung besonders relevant, da hier Personen an Praktiken in einem durch legale und organisationale Faktoren vorstrukturierten System teilnehmen (Hädicke und Wiesemann 2021). Peña-Guzmán und Reynolds (2019) vertreten die Auffassung, dass ableistische Netzwerke sozialer Bedeutung die medizinische Versorgung von Menschen mit psychischen Erkrankungen strukturieren. Im folgenden Abschnitt zeigen wir, inwiefern Ableismus mit einem besonders hohen Risiko für testimoniale Ungerechtigkeit gegenüber Menschen mit psychischer Erkrankung einhergeht.

### Ableistische Netzwerke sozialer Bedeutung

„Ableismus“ bezeichnet ein Netzwerk sozialer Bedeutung, in dem ein(e) perfekte(r), idealtypische(r) Körper und Psyche kulturell konstruiert werden, die als essentiell und vollständig menschlich projiziert werden (Campbell 2001). Körperliche und psychische Gesundheit sowie die Abwesenheit von Beeinträchtigungen werden damit als deskriptive und präskriptive Norm gesetzt (Peña-Guzmán und Reynolds 2019). Rashed (2019) zufolge werden Abweichungen von psychischen Normen im medizinischen Modell als inhärent behindernd angesehen. Demgegenüber postulieren soziale Modelle von Behinderung, dass Behinderung primär und maßgeblich durch die Beschaffenheit der sozialen Umwelt verursacht wird. Zum Beispiel vertritt die Nutzer*innen-Organisation *Hearing Voices Network* die Position, dass es nicht notwendigerweise den Alltag einschränkt, Stimmen zu hören, sondern dass eher der soziale Ausschluss aufgrund nicht normativen Verhaltens Behinderung produziert. Peña-Guzmán und Reynolds (2019) sowie Campbell (2009) argumentieren, dass ableistische Netzwerke sozialer Bedeutung einen epistemischen Rahmen bilden, der medizinische Praktiken, wie etwa die ärztliche Interaktion oder Prozesse des Diagnostizierens, strukturiert. Empirische Untersuchungen zeigen, dass das Vorliegen einer (psychischen) Behinderung dazu führt, dass unabhängig von tatsächlichen kognitiven Fähigkeiten eher *über* die betroffene Person gesprochen wird als *mit* ihr, dass eher begleitende Personen angesprochen werden oder dass die Person mit Behinderung infantilisiert wird (Gibbs et al. 2008; Smeltzer et al. 2012).

### Testimoniale Ungerechtigkeit und psychische Erkrankung

Ableistische Netzwerke sozialer Bedeutung führen zu einer Abwertung der epistemischen Kompetenzen von Personen, deren Sprech- und Denkweise nicht dominanten Normen entsprechen. Dies betrifft auch Menschen mit psychischer Erkrankung, die nach wie vor starker gesellschaftlicher Stigmatisierung unterliegen (Angermeyer et al. 2013; Henderson et al. 2014). So kodieren Stereotype des „Psychisch Kranken“ oder „Verrückten“ eine Abweichung von rational-normativen Denkmustern als defizitär (Scrutton 2017; Crichton et al. 2017). In der Folge wird Menschen mit einer psychischen Erkrankung häufig vorurteilshaft Glaubwürdigkeit und Intelligenz abgesprochen (Jongsma et al. 2017). Darum haben verschiedene Autor*innen darauf hingewiesen, dass Menschen mit psychischer Erkrankung einem besonders hohen Risiko für testimoniale Ungerechtigkeit unterliegen (Sanati und Kyratsous 2015; Dohmen 2016; Kurs und Grinshpoon 2018).

Dies betrifft auch verschiedene medizinische Settings. So zeigen Studien aus dem Bereich der Stigma- und Diskriminierungsforschung, dass neben der Allgemeinbevölkerung auch medizinisches Personal, sowohl in der somatischen als auch in der psychosozialen Gesundheitsversorgung, bewusst und unbewusst negative Stereotype gegenüber Menschen mit psychischen Erkrankungen hat (Thornicroft et al. 2007; Angermeyer et al. 2013; Henderson et al. 2014; Knaak et al. 2015). Diese Stereotype beinhalten Annahmen, dass Personen aufgrund psychischer Erkrankung nicht zuverlässig an gemeinsamen epistemischen Prozessen teilnehmen können. Wie empirische Untersuchungen zeigen, kann dies im psychiatrischen Setting zur Folge haben, dass Psychiater*innen Menschen mit schwerer psychischer Erkrankung, wie Depression, eine partizipative Entscheidungsfindung nicht zutrauen (Zisman-Ilani et al. 2021).

Auch im somatischen Setting beeinflusst die Kenntnis über das Vorliegen einer psychiatrischen Diagnose klinische Prozesse maßgeblich, mitunter unabhängig von der tatsächlichen aktuellen psychischen Belastung. Mitarbeitende verschiedener Notaufnahmen berichteten in einer qualitativen Studie, dass Beschwerden über körperliche Symptome häufig ohne ausführliche Diagnostik kausal vorliegenden psychiatrischen Diagnosen zugeordnet wurden (Shefer et al. 2014). Ähnliches berichteten auch an Diabetes mellitus erkrankte Personen mit schwerer psychischer Erkrankung:

What I find is people ignore my symptoms because I’ve got a mental health problem. … because in hospitals mental health, the minute that word is mentioned it’s like you’re suddenly nothing, a nobody, you’re like, you know whatever you’re saying you’re lying or you’re faking it or you’re just a waste of time, wasting their time. (Nash 2014, S. 719)

Negative Stereotype über die epistemische Unzuverlässigkeit von Menschen mit psychischer Erkrankung bleiben verbreitet, obwohl Betroffenenverbände, Aktivist*innen und Theoretiker*innen auf die epistemisch privilegierte Position von Betroffenen als Spezialist*innen ihrer Erkrankung hinweisen (Scrutton 2017; Drożdżowicz 2021). Auch im geschilderten zweiten Fall scheint, dass die Nutzerin sich epistemisch in der besten Position befindet, die neu aufgetretenen Bauchschmerzen von den ihr vorbekannten Stress-assoziierten Beschwerden zu unterscheiden. Dennoch wird ihrer Aussage weniger Bedeutung zugesprochen als bei einer Person ohne psychiatrische Diagnose zu vermuten wäre. In beiden beschriebenen Fällen werden somatische Symptome der vorbekannten psychiatrischen Diagnose zugeordnet. Dabei werden die Personen durch das medizinische Personal aufgrund ihrer psychischen Erkrankung als unfähig erlebt, körperliche Symptome wahrzunehmen, ihre Relevanz einzuschätzen und diese zuverlässig mitzuteilen. Mit Hookway (2010) kann dies als ein* informational prejudice*, ein Vorurteil gegenüber der Fähigkeit einer Person, für einen epistemischen Prozess relevante Informationen bereitzustellen, beschrieben werden. Das auf Stereotypen basierende negative Glaubwürdigkeitsurteil ist vorurteilshaft, da es ohne weitere Prüfung vorhandener Evidenz, wie einer genauen klinischen Anamnese und körperlichen Untersuchung, erfolgt. Somit handelt es sich um Fälle testimonialer Ungerechtigkeit. Wie Kidd (2019) argumentiert, sollten Stereotype und ihr Einfluss auf Glaubwürdigkeitsurteile intersektional, d. h. in einem komplexen Zusammenspiel mit weiteren sozial bedeutsamen Gruppenzuschreibungen, zum Beispiel über sexistische und rassistische Stereotype, untersucht werden. Im zweiten Fall können auch die oben genannten gegenderten Stereotype über weiblichen Schmerz hinzukommen und das negative Glaubwürdigkeitsurteil verstärken.

## Ethische Analyse

### Primärer Schaden

Im Folgenden analysieren wir testimoniale Ungerechtigkeit unter ethischen Gesichtspunkten mit einem besonderen Fokus auf das Setting der Gesundheitsversorgung. Fricker analysiert die Herabwürdigung der Glaubwürdigkeit einer Person aufgrund einer negativen Stereotypisierung als moralisch und epistemisch problematisch. Es ist primär moralisch problematisch, da einer Person *qua* ihrer zugeschriebenen sozialen Identität eine zentrale menschlichen Eigenschaft, nämlich die einer*s epistemische*n Agent*in, abgesprochen wird (Fricker 2007, S. 44). Dabei handelt es sich um einen intrinsischen moralischen Schaden, da eine Person durch den Ausschluss als epistemische*r Agent*in als Person abgewertet wird.^4^

### Folgeschäden

Darüber hinaus können durch testimoniale Ungerechtigkeit unterschiedliche Folgeschäden entstehen. Erstens kann testimoniale Ungerechtigkeit negative Konsequenzen auf das Vertrauen betroffener Personen in ihre eigenen epistemischen Kompetenzen haben (Fricker 2007, S. 54). Dies ist besonders problematisch und ethisch relevant, da Menschen mit psychischer Erkrankung aufgrund ableistischer Normen im Umgang mit ihren eigenen kognitiven Fähigkeiten verunsichert sein können.

Zweitens kann testimoniale Ungerechtigkeit zu einem Vertrauensverlust in das medizinische System führen. Denn testimoniale Ungerechtigkeit kann mit hohen Latenzzeiten bis zur Diagnosestellung, zeitintensiven Ärzt*innenbesuchen, Fehldiagnosen und -behandlungen und dem „Trauma, nicht gesehen zu werden“ verbunden sein (Hedva 2020, S. 29). In der Folge können sich Menschen vom medizinischen Gesundheitssystem abwenden. Eine reduzierte Inanspruchnahme des Gesundheitssystems ist wiederum mit einem schlechteren Gesundheitszustand verbunden.

Als weitere Folge des Vertrauensverlustes können Menschen mit psychischer Erkrankung bestimmte Informationen absichtlich zurückhalten. Dotson (2011) beschreibt, dass Menschen, die häufig erleben, dass ihre Berichte angezweifelt, ignoriert oder übergangen werden, ihre eigenen Aussagen an die erwartete negative soziale Reaktion der Zuhörer*innenschaft anpassen. Beim sogenannten *testimonial smothering* zensiert eine Person vorsorglich selbst ihre Sprechinhalte, so dass diese nur noch Inhalte enthalten, für die die Zuhörer*innenschaft kommunikative Kompetenz zeigt. Wenn eine Person wiederholt die Erfahrung macht, dass das medizinische Personal nicht adäquat auf ihre kommunikativen Bemühungen eingeht und kein sicheres Umfeld zur Kommunikation ihrer Anliegen schafft, kann dies dazu führen, dass sie es unterlässt, somatische Symptome zu schildern:

Within the health care setting, this testimonial smothering can manifest as patients choosing not to disclose information about themselves, their symptoms, and their medical history, because they believe that the information will be either ignored or misinterpreted by their physician who they perceive to be negatively stereotyping them. (Puddifoot 2019, S. 77–78)

Drittens kann durch testimoniale Ungerechtigkeit ein epistemischer Folgeschaden verursacht werden, da an die zuhörende Person gerichtete Informationen nicht empfangen oder, im Falle vom *testimonial smothering*, zurückgehalten werden (Fricker 2007, S. 43). In medizinischen Settings kann dieser Informationsverlust zu vermeidbaren medizinischen Behandlungsfehlern führen. Peña-Guzmán und Reynolds (2019) subsumieren so entstandene medizinische Fehler unter der Kategorie „schematischer Fehler“, da sie durch die Anwendung eines verzerrten Netzwerks sozialer Bedeutung entstehen. Die Besonderheit besteht darin, dass alle relevanten Informationen zur Vermeidung medizinischer Fehler verfügbar wären, jedoch nicht angemessen interpretiert, umgesetzt oder kommunizierbar gemacht werden.

Hier könnte eingewendet werden, dass manche Stereotype angemessen sind, indem sie auf statistischen Verteilungen beruhen. Folglich könnte ihre Anwendung bei der Einschätzung von Symptomen bei medizinischen Entscheidungen unter limitierten zeitlichen Ressourcen epistemisch gerechtfertigt sein. Puddifoot (2019) argumentiert demgegenüber, dass auch die Anwendung von statistisch korrekten Stereotypen zu epistemischen Fehlern führen kann. Grund ist, dass Stereotype zu einer Überbewertung oder einer Stereotyp kongruenten Wahrnehmung von Symptomen führen können. Dies kann den weiteren diagnostischen Prozess einengen, so dass nicht alle relevanten Differentialdiagnosen angemessene Aufmerksamkeit erfahren. Dies ist epistemisch kostspielig.

Alle Personen sind einem gewissen Risiko ausgesetzt, durch medizinische Fehler geschädigt zu werden. Jedoch führen schematische Fehler aufgrund von Ableismus für Personen mit psychischer Erkrankung zu einem disproportional hohen Risiko (Peña-Guzmán und Reynolds 2019). Folglich stellt testimoniale Ungerechtigkeit eine entscheidende Ursache für die schlechtere somatische Gesundheitsversorgung von Menschen mit psychischer Erkrankung dar.

### Diskriminierung

Schließlich handelt es sich bei testimonialer Ungerechtigkeit um Diskriminierung. Wir verstehen Handlungen, Praktiken und Gesetze als diskriminierend, wenn sie 1. auf der zugeschriebenen sozialen Gruppe einer Person beruhen, 2. die Gruppenzuschreibung sozial salient ist, indem sie die Interaktionen in sozialen Kontexten situationsübergreifend strukturiert, und 3. wenn die Handlungen, Praktiken und Gesetze zu einem Nachteil, Schaden oder Unrecht der betroffenen Person führen (Lippert-Rasmussen 2018; Altman 2020).^5^ Wir haben argumentiert, dass die Kenntnis über eine psychiatrische Diagnose im medizinischen Setting zur Gruppenzuschreibung der „psychisch erkrankten“ Nutzer*innen führt. Diese Kategorisierung strukturiert kontext-übergreifend medizinische Versorgungssettings und ist damit sozial salient. Darüber hinaus führt sie zu Nachteilen für Nutzer*innen im Sinne des primären Schadens sowie der verschiedenen Folgeschäden. Damit erfüllt testimoniale Ungerechtigkeit gegenüber Menschen mit psychischer Erkrankung *qua* ableistischer Netzwerke sozialer Bedeutung im Setting der Gesundheitsversorgung die Kriterien von Diskriminierung. Puddifoot (2018) argumentiert, dass testimoniale Ungerechtigkeit spezifisch als *epistemische Diskriminierung* verstanden werden kann. Diese ist dadurch gekennzeichnet, dass Personen aufgrund einer sozial salienten zugeschriebenen Gruppenzugehörigkeit verwehrt wird, ihre vollen Kapazitäten als epistemische Agent*innen auszuüben. Dabei ist von besonderer Bedeutung, wie sich hier epistemische Diskriminierung in einen Kontext struktureller Diskriminierung einfügt. Menschen mit psychischer Erkrankung wird durch verschiedene strukturelle Barrieren der Zugang zur Gesundheitsversorgung erschwert (Mitchell et al. 2009; Rüsch und Berger 2019). Testimoniale Ungerechtigkeit kann zu einer Reproduktion der strukturellen Diskriminierung beitragen, wenn Personen aufgrund erlebter epistemischer Diskriminierung trotz eines bestehenden Bedarfs Behandlungsangeboten fernbleiben. Mit dem Unterzeichnen der UN-Behindertenrechtskonvention (UN-BRK) hat sich Deutschland zu einem aktiven Einsatz gegen Diskriminierung von Menschen mit psychischen Behinderungen verpflichtet (Bundesregierung 2017). Laut § 25 UN-BRK ist ein vollumfänglicher Zugang zur medizinischen Gesundheitsversorgung gleicher Qualität sicherzustellen. Darum schlagen wir zuletzt Strategien zur Verbesserung der medizinischen Praxis unter ethischen Gesichtspunkten vor.

### Implikationen für die klinische Praxis

Zunächst weisen wir auf individueller Ebene auf die Wichtigkeit der Gestaltung einer tragfähigen Beziehung zwischen medizinischem Personal und Nutzer*innen hin, in der jede Person in ihrer Individualität wahrgenommen wird (Maio 2017). Eine Anerkennung aller Personen unabhängig von der zugeschrieben sozialen Gruppe oder vorliegenden Diagnosen ist Voraussetzung für die Erfüllung normativer Standards der ärztlichen Profession, wie sie zum Beispiel im Genfer Gelöbnis definiert werden (Weltärztebund 2017). Eine solche Anerkennung ist auch Teil des *Code of Ethics* der *World Psychiatric Association* (World Psychiatric Association 2020). Darum obliegt medizinisch Tätigen eine kritische Selbstreflexion. Hängen ihre professionelle Haltung, ihre Kommunikation oder klinische Urteile von der sozialen Identität von Nutzer*innen ab, ist eine Korrektur geboten. Eine solche kritische Haltung könnte der Tugend der *reflexive awareness* entsprechen, die Fricker (2007) zur Vermeidung testimonialer Ungerechtigkeit vorschlägt.

Dabei besteht eine nicht abschließend geklärte Frage darin, ob insbesondere implizite Stereotype hinreichend kontrolliert werden können. Ergebnisse aus der experimentellen Psychologie stützen jedoch die These, dass der Einfluss von implizitem Bias auf Urteilsbildungen kontrollierbar gemacht werden kann (Brownstein und Saul 2016). Zum Beispiel kann ärztliches Personal in Anti-Bias-Trainings Strategien lernen, um Nutzer*innen als Individuen, und nicht als Teil einer sozialen Gruppe wahrzunehmen (Chapman et al. 2013). So konnte durch ein Anti-Stigma-Training mit Medizin-Studierenden eine Reduktion negativer Vorurteile gegenüber Menschen mit psychischer Erkrankung erzielt werden (Wechsler et al. 2020).

Da Netzwerke sozialer Bedeutung Teil sozialer Strukturen sind und da die Auswirkungen sozialer Praktiken nicht allein auf die Intentionen und Handlungen von Individuen zurückgeführt werden können, sind Interventionen allein auf individueller Ebene unzureichend (Anderson 2012; Haslanger 2020). Auf institutioneller Ebene berücksichtigt die Organisationsethik, dass Organisationen ein von den in ihren Strukturen handelnden Personen unabhängiges Eigenleben haben, in dem soziale Praktiken und die Organisationskultur eine wichtige Rolle spielen (Wallner 2015). Zum einen ist bekannt, dass der Einfluss von impliziten Bias auf medizinisches Personal unter Zeitmangel verstärkt ist, so dass eine Veränderung von Abläufen und die Verstärkung zeitlicher und personeller Ressourcen sinnvoll erscheinen (Croskerry 2002). Zum anderen kann eine Veränderung interner Praktiken die Organisationskultur positiv beeinflussen. Mitarbeitenden-Schulungen, diskriminierungssensible und anti-ableistische Ausbildung, Super- und Intervisionen oder ein niedrigschwelliges Beschwerde- und Fehlermanagement in Kliniken können zur Korrektur diskriminierender Praktiken beitragen (Campbell 2009). Bei der Planung von anti-ableistischer medizinischer Praxis sollten im Sinne der vollen Partizipation Menschen mit psychischer Erkrankung beteiligt sein (Iezzoni 2016). Dies kann dazu beitragen, negative Stereotype über Menschen mit psychischer Erkrankung abzubauen und eine Veränderung ableistischer Netzwerke sozialer Bedeutung bewirken.

Schließlich sollte berücksichtigt werden, dass verzerrte Netzwerke sozialer Bedeutung resistent gegenüber Veränderung sind, solange sich tatsächliche gesellschaftliche Verhältnisse nicht verändern. Dies könnte erklären, warum trotz verschiedener Erfolge von Betroffenen-Initiativen, wie der Verbesserung der psychischen Gesundheitsversorgung mittels innovativer Modelle oder der Verankerung von Rechten von Menschen mit Behinderung, Stigma und Vorurteile gesellschaftlich stabil bleiben (Angermeyer et al. 2013). Solange Menschen mit psychischer Erkrankung und Behinderung von einer vollen gesellschaftlichen Partizipation ausgeschlossen sind, zum Beispiel aufgrund von diskriminierender Gesetzgebung oder diskriminierenden Einstellungs- und Beförderungspraktiken (von Kardorff 2016), ist bloße Anti-Diskriminierung-Arbeit nicht ausreichend (Iezzoni 2016). Um ableistische Netzwerke sozialer Bedeutung nachhaltig zu verändern, müssen soziale Praktiken demnach nicht nur auf individueller und organisationaler, sondern auch auf struktureller Ebene adressiert und verändert werden.

## Footnotes

1 Aufgrund der vorliegenden Literatur beziehen sich unsere Analysen vorrangig auf den europäischen und US-amerikanischen Raum.

2 Soziale Modelle von Behinderung sind kritisch diskutiert und das Verhältnis zwischen Behinderung, Partizipation, und körperlicher Einschränkung unterschiedlich definiert worden, siehe Nordenfelt (1997) und Amundson und Tresky (2007).

3 Für Analysen von verschiedenen Formen epistemischer Ungerechtigkeit gegenüber Menschen mit psychischer Erkrankung durch einen Ausschluss von Prozessen der Wissensproduktion siehe Miller Tate (2019) oder Crichton et al. (2017).

4 Für eine ausführliche Darstellung und Diskussion siehe Fricker (2007, Kap. 2.3) und Pohlhaus (2014).

5 Siehe Hädicke und Wiesemann (2021) für eine Diskussion des Konzepts.

## References

[CR1] Altman A (2020) Discriminination. In: Zalta EN (Hrsg) The Stanford encyclopedia of philosophy. https://plato.stanford.edu/archives/win2020/entries/discrimination/. Zugegriffen: 22. Okt. 2021

[CR2] Amundson R, Tresky S (2007). On a bioethical challenge to disability rights. J Med Philos.

[CR3] Anderson E (2012). Epistemic justice as a virtue of social institutions. Soc Epistemology.

[CR4] Angermeyer MC, Matschinger H, Schomerus G (2013). Attitudes towards psychiatric treatment and people with mental illness: changes over two decades. Br J Psychiatry.

[CR5] Brownstein M, Saul J (2016). Moral Responsibility, Structural Injustice, and Ethics.

[CR6] Bundesregierung (2017) UN-Behindertenrechtskonvention: Übereinkommen über die Rechte von Menschen mit Behinderungen. https://www.behindertenbeauftragter.de/SharedDocs/Downloads/DE/AS/Broschuere_UNKonvention_KK.pdf?__blob=publicationFile&v=7. Zugegriffen: 19. Okt. 2021

[CR7] Campbell FK (2001). Inciting legal fictions: disability date with ontology and the ableist body of the law. Griffith Law Rev.

[CR8] Campbell FK (2009). Medical education and disability studies. J Med Humanit.

[CR9] Chapman E, Kaatz A, Carnes M (2013). Physicians and implicit bias: how doctors may unwittingly perpetuate health care disparities. J Gen Intern Med.

[CR10] Cooper LA, Roter DL, Carson KA, Beach MC, Sabin JA, Greenwald AG, Inui TS (2012). The associations of clinicians’ implicit attitudes about race with medical visit communication and patient ratings of interpersonal care. Am J Public Health.

[CR11] Crichton P, Carel H, Kidd IJ (2017). Epistemic injustice in psychiatry. BJPsych Bull.

[CR12] Croskerry P (2002). Achieving quality in clinical decision making: cognitive strategies and detection of bias. Acad Emerg Med.

[CR13] Dohmen J (2016). “A little of her language”: epistemic injustice and mental disability. Res Phil.

[CR14] Dotson K (2011). Tracking epistemic violence, tracking practices of silencing. Hypatia.

[CR15] Drożdżowicz A (2021). Epistemic injustice in psychiatric practice: epistemic duties and the phenomenological approach. J Med Ethics.

[CR16] FitzGerald C, Hurst S (2017). Implicit bias in healthcare professionals: a systematic review. BMC Med Ethics.

[CR17] Fricker M (2007). Epistemic injustice: power and the ethics of knowing.

[CR18] Gibbs SM, Brown MJ, Muir WJ (2008). The experiences of adults with intellectual disabilities and their carers in general hospitals: a focus group study. J Intellect Disabil Res.

[CR19] Hädicke M, Wiesemann C (2021). Was kann das Konzept der Diskriminierung für die Medizinethik leisten? – Eine Analyse. Ethik Med.

[CR20] Hall WJ, Chapman MV, Lee KM, Merino YM, Thomas TW, Payne BK, Eng E, Day SH, Coyne-Beasley T (2015). Implicit racial/ethnic bias among health care professionals and its influence on health care outcomes: a systematic review. Am J Public Health.

[CR21] Haslanger S (2017). Racism, ideology, and social movements. Res Phil.

[CR22] Haslanger S (2020). Failures of methodological individualism: the materiality of social systems. J Soc Philos.

[CR23] Hedva J (2020) Sick woman theory. Caring Structures:38–46. https://www.kunstverein-hildesheim.de/assets/04ac220f66/caring-structures_Heft-booklet-v2.pdf. Zugegriffen: 22. Okt. 2021

[CR24] Henderson C, Noblett J, Parke H, Clement S, Caffrey A, Gale-Grant O, Schulze B, Druss B, Thornicroft G (2014). Mental health-related stigma in health care and mental health-care settings. Lancet Psychiatry.

[CR25] Hoffmann DE, Tarzian AJ (2001). The girl who cried pain: a bias against women in the treatment of pain. J Law Med Ethics.

[CR26] Hookway C (2010). Some varieties of epistemic injustice: reflections on Fricker. Episteme.

[CR27] Iezzoni LI, Parekh R, Childs EW (2016). Stigma and persons with disabilities. Stigma and prejudice.

[CR28] Jongsma K, Spaeth E, Schicktanz S (2017). Epistemic injustice in dementia and autism patient organizations: an empirical analysis. AJOB Empir Bioeth.

[CR29] Juckel G (2014). Psychische Erkrankungen und ihre Auswirkungen auf Mortalität und Morbidität (I). Versicherungsmedizin.

[CR30] von Kardorff E, Scherr A, El-Mafaalani A, Gökcen YE (2016). Diskriminierung seelisch beeinträchtigter Menschen. Zur Paradoxie der fortlaufenden Diskriminierung „Ver-rückte“. Handbuch Diskriminierung.

[CR31] Kidd IJ (2019). Pathophobia, vices, and illness. Int J Philos Stud.

[CR32] Kidd IJ, Carel H (2017). Epistemic injustice and illness. J Appl Philos.

[CR33] Knaak S, Patten S, Ungar T (2015). Mental illness stigma as a quality-of-care problem. Lancet Psychiatry.

[CR34] Krones T, Marckmann G (2015). Beziehungen zwischen Patienten und Behandlungs/Betreuungsteams und gemeinsame Entscheidungsfindung. Praxisbuch Ethik in der Medizin.

[CR35] Kurs R, Grinshpoon A (2018). Vulnerability of individuals with mental disorders to epistemic injustice in both clinical and social domains. Ethics Behav.

[CR36] Lippert-Rasmussen K, Lippert-Rasmussen K (2018). The philosophy of discrimination. An introduction. The Routledge handbook of the ethics of discrimination.

[CR37] Maio G (2017). Mittelpunkt Mensch: Lehrbuch der Ethik in der Medizin: mit einer Einführung in die Ethik der Pflege.

[CR38] Miller Tate AJ (2019). Contributory injustice in psychiatry. J Med Ethics.

[CR39] Mitchell AJ, Malone D, Doebbeling CC (2009). Quality of medical care for people with and without comorbid mental illness and substance misuse: systematic review of comparative studies. Br J Psychiatry.

[CR40] Nash M (2014). Mental health service users’ experiences of diabetes care by mental health nurses: an exploratory study. J Psychiatr Ment Health Nurs.

[CR41] Nordenfelt L (1997). The importance of a disability/handicap distinction. J Med Philos.

[CR42] Peña-Guzmán DM, Reynolds JM (2019). The harm of ableism: medical error and epistemic injustice. Kennedy Inst Ethics J.

[CR43] Pohlhaus G (2014). Discerning the primary epistemic harm in cases of testimonial injustice. Soc Epistemology.

[CR44] Puddifoot K, Lippert-Rasmussen K (2018). Epistemic discrimination. The Routledge handbook of the ethics of discrimination.

[CR45] Puddifoot K (2019). Stereotyping patients. J Soc Philos.

[CR46] Rashed MA (2019). In defense of madness: the problem of disability. J Med Philos.

[CR47] Rüsch N, Berger M, Berger M (2019). Das Stigma psychischer Erkrankungen. Psychische Erkrankungen: Klinik und Therapie: in Zusammenarbeit mit der Cochrane Deutschland Stiftung.

[CR48] Sanati A, Kyratsous M (2015). Epistemic injustice in assessment of delusions. J Eval Clin Pract.

[CR49] Saul J, Kidd IJ, Medina J, Pohlhaus G (2017). Implicit bias, stereotype threat, and epistemic injustice. The Routledge handbook of epistemic injustice.

[CR50] Schicktanz S (2012). Epistemische Gerechtigkeit. Sozialempirie und Perspektivenpluralismus in der Angewandten Ethik. Dtsch Z Philos.

[CR51] Schneider F, Erhart M, Hewer W, Loeffler LA, Jacobi F (2019). Mortality and medical comorbidity in the severely mentally ill. Dtsch Arztebl Int.

[CR52] Scrutton AP, Kidd IJ, Medina J, Pohlhaus G (2017). Epistemic injustice and mental illness. The Routledge handbook of epistemic injustice.

[CR53] Shefer G, Henderson C, Howard LM, Murray J, Thornicroft G (2014). Diagnostic overshadowing and other challenges involved in the diagnostic process of patients with mental illness who present in emergency departments with physical symptoms—a qualitative study. Plos One.

[CR54] Smeltzer SC, Avery C, Haynor P (2012). Interactions of people with disabilities and nursing staff during hospitalization. Am J Nurs.

[CR55] Thornicroft G, Rose D, Kassam A (2007). Discrimination in health care against people with mental illness. Int Rev Psychiatry.

[CR56] Wallner J, Marckmann G (2015). Organisationsethik: Methodische Grundlagen für Einrichtungen im Gesundheitswesen. Praxisbuch Ethik in der Medizin.

[CR57] Wang Q, Xu R, Volkow ND (2020). Increased risk of COVID-19 infection and mortality in people with mental disorders: analysis from electronic health records in the United States. World Psychiatry.

[CR58] Wechsler D, Schomerus G, Mahlke C, Bock T (2020). Effektivität einer kontaktbasierten Kurzintervention zum Abbau stigmatisierender Einstellungen unter MedizinstudentInnen : Ergebnisse einer randomisierten, kontrollierten Studie (Effects of contact-based, short-term anti-stigma training for medical students : Results from a randomized controlled trial). Neuropsychiatr.

[CR59] Weltärztebund (2017) Deklaration von Genf. https://www.bundesaerztekammer.de/fileadmin/user_upload/downloads/pdf-Ordner/International/bundersaaerztekammer_deklaration_von_genf_04.pdf. Zugegriffen: 19. Okt. 2021

[CR60] Werner A, Malterud K (2003). It is hard work behaving as a credible patient: encounters between women with chronic pain and their doctors. Soc Sci Med.

[CR61] White AA (2014). Some advice for minorities and women on the receiving end of health-care disparities. J Racial and Ethnic Health Disparities.

[CR62] WHO (2015). Excess mortality in persons with severe mental disorders.

[CR63] WHO (2019) Mental disorders. https://www.who.int/news-room/fact-sheets/detail/mental-disorders. Zugegriffen: 19. Okt. 2021

[CR64] World Psychiatric Association (2020) Code of ethics for psychiatry. https://3ba346de-fde6-473f-b1da-536498661f9c.filesusr.com/ugd/842ec8_1d812c6b8a4f4d24878ee1db8a6376f6.pdf. Zugegriffen: 19. Okt. 2021

[CR65] Zisman-Ilani Y, Roth RM, Mistler LA (2021). Time to support extensive implementation of shared decision making in psychiatry. JAMA Psychiatry.

